# Insights into Coupled Folding and Binding Mechanisms from Kinetic Studies*

**DOI:** 10.1074/jbc.R115.692715

**Published:** 2016-02-05

**Authors:** Sarah L. Shammas, Michael D. Crabtree, Liza Dahal, Basile I. M. Wicky, Jane Clarke

**Affiliations:** From the Department of Chemistry, University of Cambridge, Lensfield Road, Cambridge CB2 1EW, United Kingdom

**Keywords:** biophysics, electrostatics, kinetics, protein dynamic, protein folding, signaling, coupled folding and binding, phi-value, protein-protein interactions, residual structure, IDP, protein electrostatics

## Abstract

Intrinsically disordered proteins (IDPs) are characterized by a lack of persistent structure. Since their identification more than a decade ago, many questions regarding their functional relevance and interaction mechanisms remain unanswered. Although most experiments have taken equilibrium and structural perspectives, fewer studies have investigated the kinetics of their interactions. Here we review and highlight the type of information that can be gained from kinetic studies. In particular, we show how kinetic studies of coupled folding and binding reactions, an important class of signaling event, are needed to determine mechanisms.

## Introduction

A cursory scan of scientific literature shows the increasing interest in the study of intrinsically disordered proteins, perhaps reflecting the discovery of the key role that disordered regions of proteins play in the central processes of recognition, cell signaling, and regulation. A more detailed analysis of the literature, however, reveals that the vast majority of this work is computational, theoretical, or structural, *i.e.* analysis and prediction of IDP^3^ abundance (1, 2) and of the structural properties of disordered ensembles and assemblies (3–5). Biophysical studies have largely been carried out at equilibrium, investigating the dynamics of these disordered states (6, 7), their binding affinities, and how modulation in structure or binding affinities translates into function (8). Here we discuss just how powerful kinetic studies of the coupled folding and binding of IDPs have proved to be. They are essential for determining the mechanisms of binding (9), and also allow us to address some of the outstanding questions in the IDP field.

## How Different are IDPs Anyway? The Importance of Experimental Conditions

**FIGURE 1.**
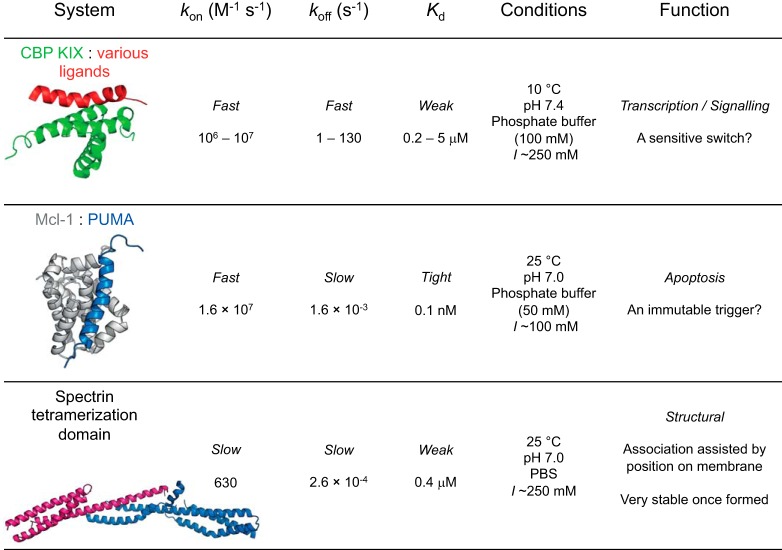
**The thermodynamic and kinetic properties of IDPs vary over orders of magnitude, and may be related to their function.** Examples are given from studies in our laboratory (21, 24, 31).

A significant proportion of proteins lack a stable, well defined, three-dimensional structure (10). These proteins, termed IDPs, can display varying amounts of residual secondary structure. Their structural heterogeneity arises from their sequence composition, which differs markedly from that of folded proteins; Gly, Pro, and charged residues are over-represented, whereas hydrophobic amino acids, which typically form the core of folded proteins, are under-represented (11–13). These compositional differences form the basis for the identification of disordered regions using bioinformatics algorithms (14). Contribution of charged residues to disorder profiles can be complex, as reflected by the importance of charge patterning in defining the extent of chain collapse (15–17). The increased conformational plasticity and altered physicochemical properties imparted by their sequence composition also change their responses to external factors such as ionic strength, temperature, and molecular crowders (18–20). Internal friction (roughness of the energy landscape) has been shown to be related to sequence composition, and may therefore be different for IDPs than denatured folded proteins (7). However, what effects do these features have when it comes to IDP-ligand interactions? Do IDPs react similarly to changes in environment as their folded counterparts? These questions can be investigated mechanistically through kinetic studies. One paradigm of early IDP studies was that disorder facilitates high specificity, low affinity binding. Although it is true that *on average* IDPs form looser complexes with faster dissociation rate constants (*k*_off_) and statistically similar association rate constants (*k*_on_) compared with folded proteins, the available range of values for both is very wide (21, 22). Thus, similar binding kinetics can be obtained for both folded and disordered proteins. It is likely that biophysical properties reflect the function of the folding and binding reaction (Fig. 1).

Electrostatic interactions can accelerate association for folded proteins by orders of magnitude (23) and cause dramatic ionic strength dependence of association rates (*k*_on_), whereas dissociation is generally affected only marginally. Electrostatic steering has also been identified for coupled folding and binding of IDPs, where *k*_on_ has been found to be beyond the expected “diffusion limit” but reduced at infinite ionic strength (24, 25). Interestingly, the electrostatic rate enhancement for c-Myb binding to KIX (CREB binding domain of CBP), and PUMA binding to Mcl-1 are under 20-fold, much less than, for example, barnase binding to barstar (about 4–5 orders of magnitude) (23). Recent binding studies utilizing NCBD demonstrated larger rate enhancements from electrostatic steering for its IDP partners when compared with its folded partners (26). It is clear that, with many IDPs having an excess of charged residues, electrostatics is of crucial relevance. Another potential difference in the role of electrostatics for disordered protein interactions is their increased propensity to undergo post-translational modifications that can alter protein charge, *e.g.* phosphorylation (27, 28). Such changes affect binding affinity (29, 30) and can be mediated both through altered long-range and local electrostatic forces and through more specific transition state effects.

Obtaining basal rate constants (*k*_on_ in the absence of long-range electrostatics) is crucial for making mechanistic conclusions on the basis of *k*_on_, as exemplified for the case of c-Myb binding to KIX (31). Here a longer version of c-Myb with increased residual structure associates faster under physiological ionic strength, suggesting that residual structure may be important in determining *k*_on._ However, the basal rate constants are identical within error, indicating that the change of charge and *not* the increase in residual helicity is responsible for the faster association at physiological ionic strength.

In addition to understanding the contribution from electrostatic interactions, kinetic studies of IDPs allow activation energies for coupled folding and binding reactions to be determined (25), giving further insight into the mechanisms by which this class of proteins achieves their functional roles. More fundamental studies of this kind are needed to determine whether IDPs really behave differently from their folded counterparts.

## Which Comes First: Binding or Folding?

**FIGURE 2.**
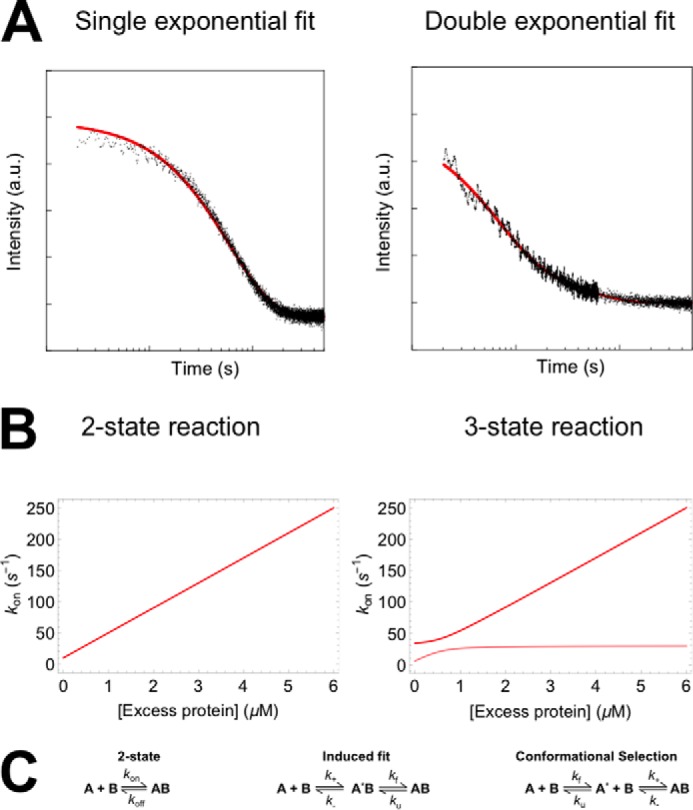
**Kinetic experiments of coupled folding and binding reactions under pseudo-first order conditions.** *A*, example kinetic traces for two-state and three-state processes, fit to single exponential and double exponential decay functions, respectively. *a. u.*, arbitrary units. *B*, dependence of observed rate constants upon protein concentrations. Analytical solutions are presented for a two-state reaction (*k*_on_ = 40 μm^−1^ s^−1^, *k*_off_ = 10 s^−1^) and a three-state IF reaction (*k*_+_ = 40 μm^−1^ s^−1^, *k*_−_ = 10 s^−1^, *k_f_* = 10 s^−1^, *k_u_* = 20 s^−1^). *C*, reaction schemes for two-state and three-state (IF and CS) processes.

Kinetics are essential to answer this question, but even when kinetics have been determined, the answer can be difficult to obtain (32–34). Practically speaking, kinetic studies involve monitoring changes in response of a probe, such as an intrinsic or extrinsic fluorophore, upon either (i) the rapid mixing of the IDP and its partner in a stopped-flow or continuous-flow format to observe complex formation (Fig. 2*A*), or (ii) sudden alteration of experimental conditions, *e.g.* temperature, leading to system relaxation back to equilibrium (35). When the reaction timescales are appropriate, it is also possible to obtain kinetics from NMR experiments (36, 37). In mixing studies, it is common to arrange the conditions such that one protein is in excess over the other (typically described as >10-fold) (32) and its concentration remains relatively constant throughout the reaction. The rate equations then become pseudo-first order and can be readily solved to obtain a description of the reaction progress with time (38). In the case of a simple two-state system, the kinetics are described by a single exponential decay function (or phase), with the associated observed rate constant being linearly dependent on the protein concentration (Fig. 2*B*). Experiments are performed at multiple concentrations, and the concentration dependence of the observed rates can then be used to extract fundamental rate constants for the system.

In coupled folding and binding studies, there is necessarily a conformational change as well as a binding step. A frequent question asked regards the order of these events. There are two extreme mechanisms that can be imagined (Fig. 2*C*). In the induced fit (IF) scheme, the IDP binds to its partner and subsequently folds. In the conformational selection (CS) scheme, the partner binds (selects) only the proteins in the IDP ensemble with the correct conformation. It can be possible to discriminate between these two situations through kinetic studies.

Unfortunately, an exact generalized description of reaction progress with time is impossible for both IF and CS schemes as there is no analytical solution to either set of rate equations. However, it is again possible to overcome this obstacle by arranging pseudo-first order conditions with the folded protein in excess, so the rate equations are simplified to linear equations that are readily solved. Kinetic traces then consist of up to two exponential decay phases (38, 40, 41) (Fig. 2*A*), which is a relatively simple functional form to fit. In both schemes, the observed rate constants and amplitudes of the two phases actually involve a defined mixture of the four fundamental rate constants, and their concentration dependences can appear very similar (39). For example, at high protein concentrations, two rate constants may be observed: one that is apparently independent of protein concentration and corresponds to the unimolecular process, and another that is linearly dependent and corresponds to the binding process. Although this can make it difficult to discriminate between the two mechanisms, the ambiguity can be cleared up by performing similar experiments under reversed pseudo-first order conditions, *i.e.* by putting the IDP in excess. If the process is IF, then the observed rate constants will remain the same, but if the process is CS, then the observed rate constants will be different (33). Indeed because it can be difficult to practically obtain an excess of A* over B,^4^ the kinetic trace may deviate from the exponential decay form in this case.

The discussion so far has described the situation when two phases are observed in kinetic traces. However, in practice, it is common to observe only one phase in these types of experiments, either because one of the rate constants is too fast, or because its amplitude is too low, to be reliably observed (25, 33, 42–44). The former may be likely in the case of IDP-partner interactions because IDPs often fold into relatively simple structures such as short α-helices upon binding, and helix (un)folding rate constants are known to be much higher than observed binding rate constants (45). Thus, we dedicate the rest of this paragraph to a discussion of the kinetic features when folding and unfolding are very fast when compared with binding and unbinding. As in these cases there is always a fixed ratio of the folded and unfolded species, *i.e.* A*B and AB, or A and A*, the folded and unfolded forms are observed as only a single species by ensemble measurements, and association kinetics display a single observed rate constant that is linearly dependent upon the protein concentration. The kinetics then reduce to the simple two-state case described previously, with the observed rate constants being related to the fundamental rate constants. For CS processes, the observed association rate constant, which is given by the gradient of the straight line, is actually significantly lower than the microscopic association rate constant. This is essentially because few of the collisions will be with “reactive” IDP protein. It has been pointed out that very fast interactions, with observed *k*_on_ > 10^7^ m^−1^ s^−1^ in the absence of electrostatic enhancement, are therefore inconsistent with CS schemes (25, 32). In contrast, the observed gradient for IF processes represents the binding rate constant and can be similar to those observed between pairs of folded proteins, whereas dissociation is slowed because it can only occur from the intermediate state. Observations so far have shown no significant differences in reported association rate constants for IDPs when compared with folded proteins (21), which might suggest that IF is the preferred mechanism; however, both cover a large range of values, and it is possible that differences in electrostatic rate enhancements are masking an effect. It is often claimed that disorder might enhance association rates of IF mechanisms, through increasing capture radius (“fly-casting”); however, this is likely to make only a small contribution (<2-fold) and has yet to be experimentally demonstrated (46, 47).

It is important to note that whereas reversing pseudo-first order conditions where two phases are reliably observed can discriminate between IF and CS, this is not always possible when (un)folding rate constants are high and the process is apparently two-state. In this case, reversing pseudo-first order conditions does not change the observed rate constant for either scheme. However, there are two circumstances where a CS mechanism is indicated. The first occurs if an observed rate constant decreases with protein concentration, which happens when conformational changes are slow when compared with unbinding (39). The second occurs if observed kinetics deviate from single exponential behavior and/or the observed rate constant obtained with the folded partner in excess. This can occur if pseudo-first order conditions have not been achieved. Because pseudo-first order conditions with A over B are easily achieved for IF, but difficult to achieve with A* over B in CS (only a small proportion of unbound IDP is folded), this behavior suggests CS.

Although the majority of kinetic studies arrange pseudo-first order mixing conditions to achieve exponential decay kinetics, in the case of a two-state system (single phase observed with no populated intermediate), it is actually possible to solve the rate equations analytically (21, 25). If it is possible to perform experiments at concentrations such that *k*_off_ makes a significant contribution to the observed kinetics, then both *k*_on_ and *k*_off_ can be estimated from a single mixing experiment (21, 25).

Dissociation kinetic experiments can also be very informative. They typically involve dilution of a labeled preformed complex into a large excess of unlabeled partner protein, which ensures virtually irreversible dissociation of the labeled version (31, 42, 43). Care must be taken in these experiments because if the concentration of unlabeled competitor is not high enough, the observed dissociation rate constant will depend upon competitor concentration and will not be accurate. For two-state reactions, where A* or A*B are not significantly populated, the ratio *k*_off_/*k*_on_ will equal the observed *K_d_* and *k*_off_ matches the *y* axis intercept in the association kinetic graph.

Finally, it is worth noting that the viewpoint of pure IF or CS mechanisms is a likely oversimplification. Processes might contain elements of both mechanisms, *e.g.* selection of partially folded IDPs in the ensemble. It is also possible that both mechanisms exist in parallel, with flux through each depending upon experimental conditions including protein concentration (48).

## What Is the Role of Order within Disorder?

Although largely unstructured, IDPs can contain regions of transient secondary structure. In the case of IDPs that undergo coupled folding and binding, the presence and abundance of the bound, folded conformation within the IDP ensemble are potentially important. For example, combining structural data from NMR with equilibrium measurements has indicated that increasing the proportion of unbound IDP with a structure that resembles the bound state enhances the binding affinity for the partner protein (34, 49). Of course, increased unbound order and enhanced complex stability are not necessarily advantageous for the function of the IDP (8).

Kinetic analysis is required to answer the key question from these studies. Is the increased complex affinity due to an enhanced *k*_on_ or reduced *k*_off_? Mechanistically, an increased *k*_on_ upon increasing the order within the unbound ensemble might indicate that the reaction is proceeding via a CS mechanism. However, care must be taken in this analysis, as an increased *k*_on_ would also be observed for IDPs where the rate-limiting folding step occurs after binding, *i.e.* the IF mechanism. Here, it is not the abundance of free structured IDP that is influencing the *k*_on_; instead, increased structure may increase the *k*_on_ by lowering the energy of the transition state for folding once bound.

A few studies have investigated the influence of residual structure on the kinetics of coupled folding and binding of IDPs. For association of c-Myb with KIX, increasing the residual structure of c-Myb, through the use of the helix stabilizer trifluoroethanol (50) or modulation of peptide length (31), decreases *k*_off_ without significantly altering *k*_on_ (suggesting an IF mechanism). It has been suggested that positive correlation of the *k*_on_ from the φ-value analysis for this system (51) with the predicted helicity indicates that the process may involve some form of CS (37) but, as described above, this apparent correlation could also be due to a lowering of the transition state barrier for folding. Mutation of surface residues in PUMA to proline, which destabilizes helices, was found to reduce its affinity for Mcl-1. Through kinetic analysis, it was shown that this reduction is due to an increase in *k*_off_, with no significant changes in *k*_on_ (52). In contrast, the enhanced affinity for the CID domain of ACTR with NCBD upon increasing residual helical propensity was due to both due to an increase in *k*_on_ and a decrease in *k*_off_ (34).

So far, most studies show an increase in affinity upon increasing residual structure. However, the differing kinetic explanations behind the increases in affinity emphasize the importance of thorough kinetic analysis in describing mechanisms.

## Probing Transition States

Analyzing an experimental system at residue level allows probing transition states or short-lived intermediates on a reaction pathway. Several studies in protein folding have applied site-directed mutagenesis along with biophysical measurements to understand folding mechanisms (53). Such mutational analysis along with kinetics can also be applied to IDPs to study interactions with their partners in more detail.

Why is it important to look at transition states? Interactions between IDPs and their partners are complex reactions. The NMR techniques used to study these interactions can identify the unbound disordered IDP, the fully bound complex structure, and in some cases, stable intermediates (36, 37). However, it is particularly important to visualize the unpopulated transition states to understand the critical molecular contacts formed during these coupled folding and binding reactions. This can only be achieved through φ-value analysis, which maps the structure formation in the transition state by comparing rate constants for wild-type and mutant proteins (54).

In protein folding studies, folding and unfolding rate constants are used to calculate φ-values. Analogously, for an IDP system, kinetic rate constants (*k*_on_ and *k*_off_) and *K_d_* are used to calculate the φ-values using Equation 1.

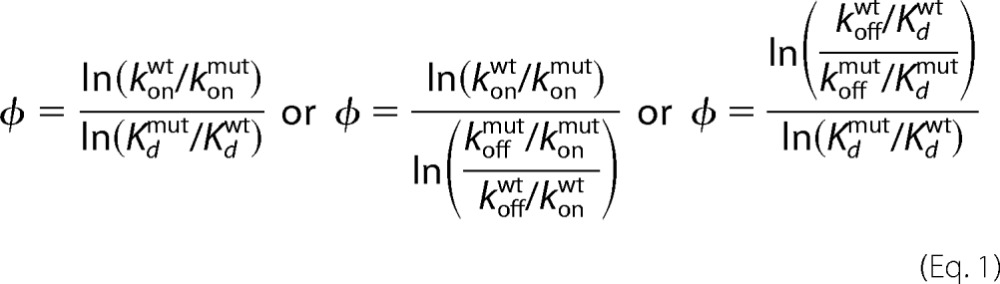


**FIGURE 3.**
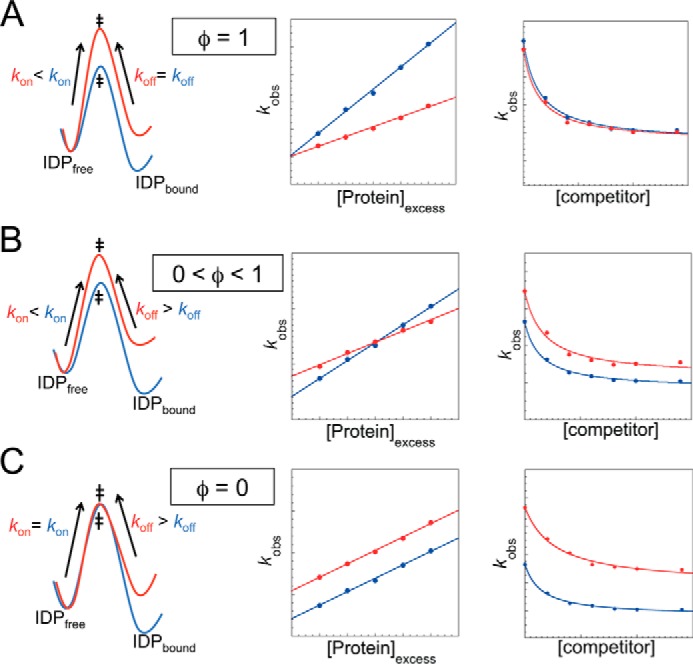
**Relationship between association and dissociation rate constants and φ-values for apparent two-state systems.** Shown are energy diagrams (*first column*), observed association rate constants under pseudo-first order conditions (*middle column*), and observed dissociation rate constants (*third column*) for wild-type IDP (*blue*) and mutant IDP (*red*). *A*, φ = 1, *i.e.* native interactions are formed in the transition state. *k*_on_ is lower, and *k*_off_ is unchanged. *B*, 0 < φ <1 structure is partially formed, resulting in changes in both *k*_on_ and *k*_off_. *C*, φ = 0, residue is as unstructured at the transition state as in the unbound state. *k*_on_ is unchanged, and *k*_off_ is increased. The rate constants *k*_on_ and *k*_off_ are controlled by energy barrier sizes (*first column*), and are determined from straight-line gradients in association mixing experiments (*second column*) and from high concentration asymptotes in out-competition dissociation mixing experiments (*third column*), respectively.

Point mutations in a protein may change *k*_on_ and *k*_off_ as shown in Fig. 3. The φ-value for each residue reports on the proportion of intermolecular or intramolecular native contacts it makes at the transition state. Where φ = 1, these contacts are fully formed (Fig. 3*A*). Where φ = 0, these contacts are as unformed in the transition state as they are in the unbound state (Fig. 3*C*). Intermediate φ-values reflect intermediate structure formation (Fig. 3*B*). Particular care has to be taken in interpreting values of association reactions because early contacts may be non-native (43). Conventionally, interfacial residues are mutated to Ala to probe for hydrophobic interactions (tertiary structure), and surface-exposed residues are mutated to Ala and Gly to probe for helix formation (secondary structure). Care must be taken if charged residues are mutated because, as we have seen, *k*_on_ values are particularly sensitive to changes in electrostatics.

**FIGURE 4.**
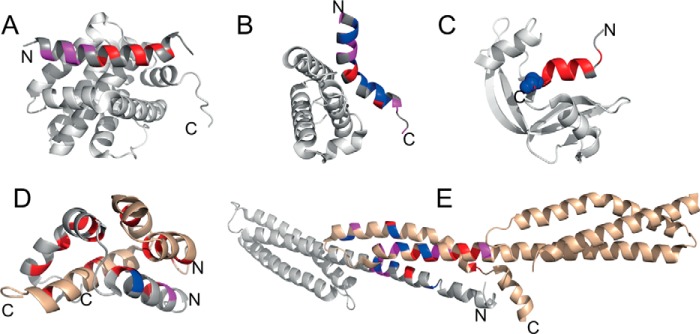
**Illustrations of coupled folding and binding reaction transition states.** φ-Values are mapped onto structures of the following complexes: *A*, PUMA·Mcl-1 (PDB: 2ROC) (43); *B*, c-Myb·CBP KIX (PDB: 1SBO) (51); *C*, S-peptide·S-protein (PDB: 1FEV) (55); *D*, ACTR·NCBD (PDB: 1KBH) (56; *E*, α·β spectrin tetramerization domain (PDB: 3LBX) (44). In *A*, *B*, and *C*, the folded partners are shown in *gray*. In *D* and *E*, both partners are disordered; one is shown in *gray*, and one is shown in *bronze*. The residues in *blue*, *magenta*, and *red* represent high (φ > 0.6), medium (0.25 ≥ φ ≤0.6), and low (φ < 0.25) φ-values, respectively. *N* and *C* denote the N and C termini of the IDP (note that in *E* the disordered regions are capped by folded domains).

The few examples in the literature where φ-value analysis has been applied to IDPs are shown in Fig. 4. In some cases, the IDP appears to be largely or partly unstructured at the transition state. For PUMA·Mcl-1, mutations to probe helix formation and hydrophobic interactions resulted in generally low φ-values, with values increasing slowly toward the N terminus, suggesting that the IDP has only embryonic structure at the N terminus (43). Low φ-values were also observed for helix formation and hydrophobic interactions in the S-protein·S-peptide system, although we note that this is not an evolved folding upon binding system, so that the general principles of association may not be the same (55). For NCBD·CID-ACTR, low φ-values were observed for intermolecular interactions, whereas higher φ-values were found for the N-terminal helix of both NCBD and CID-ACTR. Thus, although some structure is present at the N-terminal helices, the native hydrophobic interactions form after the rate-limiting transition state (56). In contrast, high φ-values were calculated for both the C terminus and the N terminus of c-Myb, implying that considerable native interactions are present in the transition state (51), perhaps surprising given that no change in *k*_on_ was seen upon increasing residual structure (31, 50). Finally, analysis of the formation of the spectrin tetramerization domain from two disordered peptides revealed high φ-values in the C terminus of helix A and the N terminus of helix B. For helix C, tertiary φ-values were higher than for helix A and B. A mechanism was proposed whereby preformed helix C provides a template onto which helix A and B dock, thus allowing core contacts to form and further folding to proceed after binding (44).

In all of these studies, a general trend of binding before folding is inferred for the coupled folding and binding reactions. Because there are few studies so far, it is not possible to come to a general conclusion about the mechanism of coupled folding and binding for IDPs. It is likely, as in protein folding (57), that there will be a spectrum of folding upon binding mechanisms, but where the interaction is very rapid, binding before folding seems, at present, to be most likely.

## Do Folded Partner Proteins Play a Role?

Coupled folding and binding studies have tended to focus on IDPs, with less attention paid to folded partner proteins. Nevertheless, as we now describe, the studies that have been performed have indicated that they may have an important role to play. Truncations in the binding interface of the folded partner protein Mcl-1 reduce the affinity for the IDP PUMA, due to an increase in *k*_off_; however, an unexpected increase in *k*_on_ occurs for some residues. Although beneficial for affinity of the complex, these residues are effectively inhibiting association. Spatial patterning of the association-inhibiting residues, together with analysis of the NMR ensemble of free Mcl-1, suggests that the hydrophobic binding grove of Mcl-1 undergoes a conformational rearrangement while binding PUMA (43). Closing of the grove around PUMA helps to maintain the complex.

The folded KIX domain of CBP is able to bind multiple transcription factors *in vivo* (58), most of which are intrinsically disordered. Several studies noted positive cooperativity between ligands binding to its two binding sites (31, 59), although the mechanism behind the cooperativity was not initially clear. Kinetic analysis revealed that both *k*_on_ and *k*_off_ were reduced when a ligand was already bound to the alternate site, and that the stabilization of the ternary complex was because the reduction in *k*_off_ exceeded that for *k*_on_ (31, 60). A similar finding was reached independently using Gō-like molecular dynamics simulations (61). Combined with NMR data showing a stiffening of the CBP KIX backbone upon ligand binding (62), this leads to the suggestion that binding of one ligand to CBP KIX changes the flexibility of the folded domain, reducing the entropic cost for ligand binding to the alternate site (31, 61). Dynamics in the folded CBP KIX domain are therefore an important factor that is able to influence the binding of its IDP partners.

These two examples demonstrate the importance of structural and dynamical changes in the folded partner protein upon ligand (IDP) binding. Further kinetic studies will help to uncover whether these findings are system-dependent or widespread.

## Conclusions and Outlook

IDPs have emerged as an important class of proteins. Their predicted abundance within the eukaryotic proteome has raised several questions. What are the advantages and disadvantages of being disordered? Why are IDPs more prominent in some processes than others? What is the functional relevance of disorder? Answering these questions is important in understanding IDPs at a fundamental and applied level, *e.g.* protein or drug design. Studies of IDPs have revealed that the conformational ensemble can be altered by external factors (*e.g.* salts, crowders), which must be taken into account when investigating coupled folding and binding reactions (43, 44, 51, 55, 56). Although more studies are required, the few that have been published indicate that the transition state of coupled folding and binding reactions is relatively unstructured. Nevertheless, residual structure appears to be an important factor that is able to influence complex affinity by modulating association and dissociation rate constants. Due to their prominence in cell signaling, IDPs have arisen as important biomedical targets. When compared with a folded protein, IDPs typically lack accessible binding pockets, making them more difficult to target with traditional small molecules. Development of new therapeutic strategies requires a thorough mechanistic understanding of coupled folding and binding reactions. Should the target be the unbound IDP, the partner protein, or the complex? Which rate constants should be altered to modulate binding affinities during therapeutic development? Through understanding the importance of electrostatics, residual structure, transition state interactions, and partner proteins, kinetic analysis can describe fundamental properties of IDPs, as well as their coupled folding and binding interactions.

## Footnotes

4 The following designations are used throughout and correspond to the schemes shown in Fig. 2*C*: A*, binding-competent IDP; B, folded partner protein; A*B, bound intermediate; AB, bound complex; A, unbound IDP. Note that in the conformational selection scheme, species A does not include unbound IDP that is binding-competent.

## Footnotes

3 The abbreviations used are:

IDP: intrinsically disordered protein
CBP: CREB-binding protein
CREB: cAMP-responsive element-binding protein
CS: conformational selection
IF: induced fit
Mcl-1: myeloid cell leukemia 1
NCBD: nuclear coactivator binding domain of CBP
PUMA: p53 up-regulated modulator of apoptosis.
